# Astrovirus infection alters gut microbial communities in a widespread neotropical bat across human-modified landscapes

**DOI:** 10.1186/s12866-026-05455-0

**Published:** 2026-07-29

**Authors:** Stefan D. Brändel, Dominik W. Melville, Kerstin Wilhelm, Victor M. Corman, Rachel Page, Christian Drosten, Marco Tschapka, Simone Sommer, Wasimuddin

**Affiliations:** 1 Institute of Evolutionary Ecology and Conservation Genomics, University of Ulm, Albert-Einstein Allee 11, Ulm, D-89069 Germany; 2 Smithsonian Tropical Research Institute, Ancon, Balboa, Apartado 0843–03092 Republic of Panama; 3 Institute of Virology, Charité Universitätsmedizin Berlin, Corporate Member of Free University, Humboldt-University and Berlin Institute of Health, Berlin, Germany; 4 German Centre for Infection Research (DZIF), Berlin, Germany; 5 Food Safety and Animal Health Research, Norwegian Veterinary Institute, Ås, Norway

## Abstract

Astroviruses are becoming a growing concern in public and veterinary health. In humans, astrovirus infections can cause severe diarrhea and may lead to neuropathological encephalitis, whereas in wildlife, these enteropathogenic viral infections often lack overt symptoms and thus remain unnoticed. Yet their close interaction with the host’s gastrointestinal microbiome might drive cascading effects with disadvantages for host health. Bats harbor many zoonotic viruses without showing signs of disease, and many species move freely along the gradient from pristine to agricultural landscapes. To better understand the impact of astrovirus (AstV) infection under a One Health framework, we investigated the gut microbiome of naturally AstV-infected Seba’s short-tailed bats (*Carollia perspicillata*, *n* = 234) inhabiting old-growth lowland forests or forest fragments embedded in an agricultural matrix in Panama. AstV prevalence was higher in forest fragments. We observed that AstV infection is associated with a shift in microbial beta but not alpha diversity, which points towards the replacement of common gut microbial taxa when infected. Indeed, potentially beneficial bacteria, such as *Lactococcus*, decreased in abundance, whereas potentially pathogenic bacteria from the *Helicobacter* genus increased in AstV-positive bats. Two *Helicobacter* haplotypes closely related to avian *Helicobacter* species were identified. We conclude that even though the impact of infection on the microbiome was not amplified in forest fragments, the higher infection likelihood in landscapes altered by humans implies more frequent or prolonged health repercussions for bats.

**Supplementary Information:**

The online version contains supplementary material available at 10.1186/s12866-026-05455-0.

## Introduction

Astroviruses (AstVs) are positive-sense, single-stranded enteric RNA viruses with an incredibly broad host range across diverse avian and mammalian hosts, including humans, and are directly and easily transmitted via the fecal-oral route [1, 2]. The recent discovery of numerous novel AstV strains, their large genetic diversity and ability for cross-species recombination highlight the risk of interspecies or even zoonotic transmission of AstVs [3–6]. In wildlife, AstV infections often persist asymptomatically, whereas in humans, infections can cause severe diarrhea and may lead to neuropathological encephalitis [7]. Symptoms of AstV infection vary depending on the age and immunological status of infected hosts with children and the immunosuppressed being most affected [1].

Land-use changes and, as a consequence, increased likelihood of human-wildlife contact amplifies the chances of disease spread beyond species boundaries [8, 9]. Food and nutrient scarcity in man-made landscapes increases overlap between wildlife, domesticated animals and humans [10–12]. Prominent examples include the regular spillover events of the zoonotic Nipah and Hendra viruses from fruit bats to pigs and horses [11, 13], and the repeated transmission of morbilli- or lyssaviruses from vampire bats to pigs and cattle [14, 15]. The high degree of population fragmentation in mosaic landscapes and the frequent crossover with novel hosts may even accelerate virus evolution, exacerbating their zoonotic risk [16, 17]. Besides, the health consequences for wildlife inhabiting fragmented forests, agricultural landscapes or urban environments are often poorly understood or entirely unknown [18–21].

The negative health effects from enteropathogenic viral infections, such as those caused by AstVs, may arise from cascading effects on the host’s gastrointestinal microbiome, enhancing viral activity or facilitating co-infections (e.g., norovirus [22], rotavirus [23], coronavirus [24], adenovirus [25], and astrovirus [26–29]). The gastrointestinal microbiome is an incredibly complex and highly diverse ecosystem, with important roles in nutrient acquisition (e.g., vitamins; [30]), toxin degradation [31] and metabolism [32] as well as in the defense against enteropathogens [33], and the development and maintenance of immune responses [34–36]. Abnormal shifts in its diversity and composition, beyond the normal range of intraspecific variation [37], could be either cause or consequence of the susceptibility to pathogens or chronic inflammatory diseases [38–41]. For instance, increased viral load and co-infections amplify gut microbial dysbiosis in bats and voles [42, 43]. The interaction between viruses and the hosts’ microbiome is an essential, yet often overlooked, component in the holobiont machinery [44–46].

Public health concerns about AstVs have prompted studies on their prevalence in bats, uncovering a high diversity of AstVs worldwide [47–50]. Due to their high prevalence and presumed mild pathogenicity, AstVs represent a perfect study system to understand potential health effects in visibly symptomless bats. At the same time, bats are seen as a model system to investigate the role of microbes in host evolution, physiology and fitness as they harbor a diverse set of microbiomes likely reflecting their ecological and phylogenetic diversity ([51] but see [52] and discussion in [53]). It is therefore unsurprising that a previous study identified AstV-mediated disruption of the gut microbial community in the Jamaican fruit bat (*Artibeus jamaicensis*) [28]. Little is known, however, about the health consequences of land-use changes for bats [54–56], and the role of disease ecology in that context. From a One Health perspective, a better understanding of how land-use change, host microbial health and infection ecology interact is pivotal to predict and prevent spillover events, and safeguard human and animal wellbeing [57].

In this study, we investigated the presence and effect of AstV infection and its interplay with land-use change on the gut microbiome diversity and composition in a neotropical frugivorous habitat generalist, the Seba´s short-tailed bat (*Carollia perspicillata*). The species is abundant throughout Central and South America, often profiting from disturbed areas and the abundance of fruits of early succession plants found in human-modified habitats, and were even observed to roost close to rural settlements [58, 59]. Even though no report to date exists about AstVs infecting *C. perspicillata*, AstVs infect other Phyllostomids (e.g [60]). Because the gut microbiome serves as the first line of defense and is closely linked to physiological stress responses, we hypothesized that AstV infections in *C. perspicillata*, particularly under human disturbance, reduce commensal bacteria and therefore alpha diversity, shift beta diversity through stochastic processes, promoting the emergence of opportunistic pathogens. Age may also play a role, as shown in *A. jamaicensis* [28], where immunologically naïve subadults exhibited altered gut microbiota when infected with AstVs. Such age-dependent responses may be common in bats. Moreover, we predict that the effect on the microbiota is amplified in AstV-positive bats inhabiting forest fragments embedded in an agricultural matrix.

## Methods

### Focus species and fecal sample collection

**Fig. 1:**
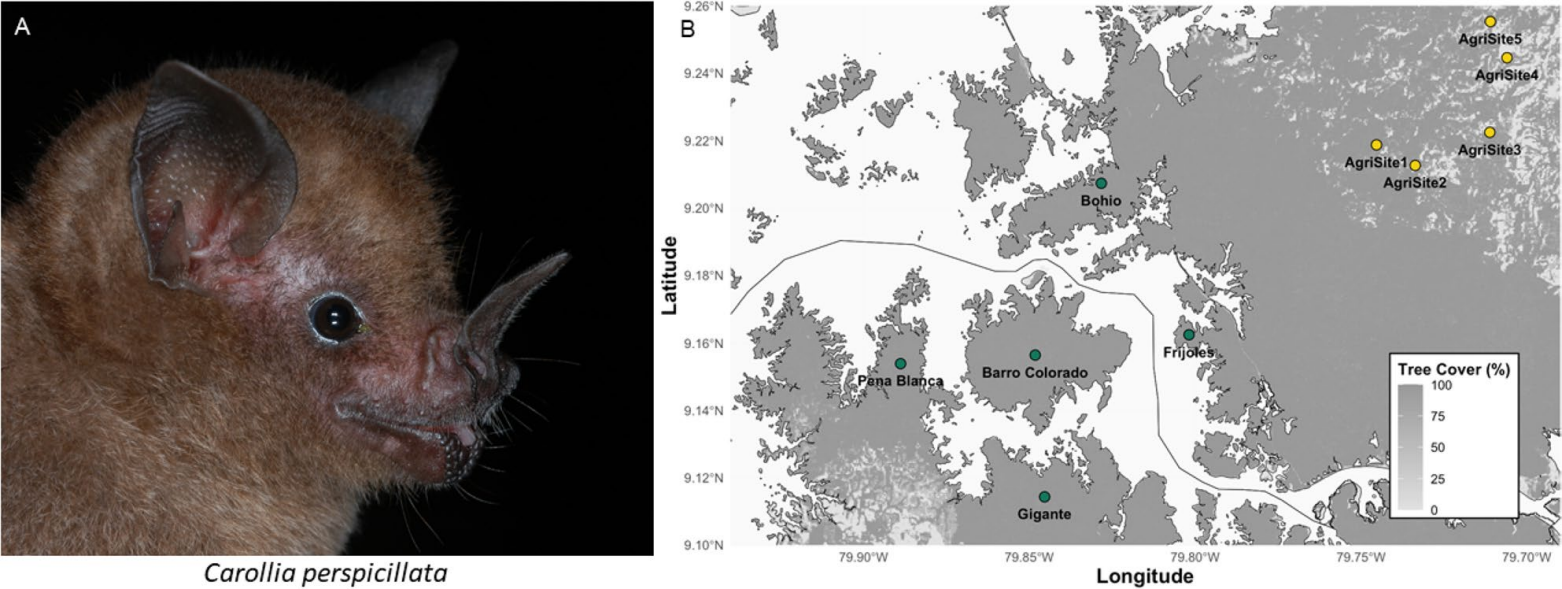
Study species and location of study sites in central Panama along the Panama Canal. **A** Image of the Neotropical frugivore *Carollia perspicillata* (credit: Marco Tschapka); (**B**) Location of study sites in the continuous lowland rainforest (green) and in forest fragments (yellow) embedded in an agricultural matrix

The phyllostomid bat *C. perspicillata* is a widespread frugivore (Fig. 1A), found in high abundance across large parts of Central and South America [61]. *C. perspicillata* consumes fruits of early successional plants, e.g., pepper plants (Family: *Piperaceae*, genus: *Piper*) [62–64] but is also known to opportunistically eat other fruits [62] and arthropods [65]. The species shows flexible roosting behavior in crevices and hollows, while it searches for food in relative proximity to its roost (mean: 1.6 km) [58, 66, 67]. Its diet preference for fruits from successional plants found frequently in agricultural landscapes cleared for livestock not only allows the species to persist but even thrive in human-disturbed habitats [58].

Bats were caught in lowland tropical rainforest ecosystems of Central Panama using mist-nets (ECOTONE, Gdynia, Poland) in a standardized sampling design at five independent sampling sites in three landscapes varying in degree of fragmentation and human disturbance (Fig. 1B): natural lowland forest, forest fragments embedded in an agricultural matrix, and forested islands surrounded by water [58]. However, *C. perspicillata* was rarely found on islands in our study and others [58, 68, 69]. Thus, the work here only concerns sites in old-growth forest and forest fragments surrounded by agricultural landscapes. After capture, individuals were sexed, and age was determined based on the ossification of the epiphyses of the digits [70]. Individual fecal samples were collected directly from bats upon defecation during handling or collected out of the fabric bags, in which bats were kept temporarily before processing, and immediately preserved in RNAlater (Life Technologies). All bats were released on site right after handling. Capture and sampling techniques followed Panamanian protocols (MiAmbiente, República de Panamá: SE/A-75-13 to SE/A-28-17) and were ethically certified by the Smithsonian Tropical Research Institute (IACUC protocols: 2014-0101-2016 to 2016-0627-2019).

### Astrovirus testing

Viral RNA was extracted from 434 fecal samples using the MagNA Pure 96 DNA and the Viral NA Small Volume Kit (Roche) corresponding to the manufacturer’s guidelines. AstV prevalence was determined by the broadly reactive nested reverse transcription-PCR (RT-PCR) assay, as described previously [28].

### Bacterial DNA extraction and 16 S rRNA gene bacterial amplicon sequencing

Microbiome analyses were carried out in a total of 234 individuals, targeting at least 30,000 raw reads per sample before quality filtering in a single sequencing run. After homogenization of fecal samples (SpeedMill PLUS Homogenizer, Analytik Jena, Germany), bacterial genomic DNA was extracted applying the NucleoSpin 96 Soil kit (Macherey-Nagel, Germany) following the manufacturer’s instructions. The universal 16 S primers 515 F/806R were used for PCR amplification of the hypervariable V4 region (291 bp) of 16 S rRNA – a standard primer pair, which leads to very high bacterial diversity and performed best in a recent primer comparison experiment [71]. We used the Fluidigm System (Access Array™ System for Illumina Sequencing Systems, ©Fluidigm Corporation) for primer tagging. PCRs (15 µl reaction volume) were executed as described previously [28, 72]. After purification (NucleoMag bead-based size selection, Macherey-Nagel, Germany) and quantification of barcoded samples (DropSense, Trinean, US), we applied paired-end sequencing on the Illumina^®^ MiSeq platform. The run included six controls.

### Bioinformatics

Bioinformatic and statistical analyses were performed within R (v4.4.1 [73]). We applied the *DADA2* pipeline [74]. Reads were trimmed from both ends to remove low-quality regions, and after denoising and merging, chimeric sequences were removed, following the ‘consensus’ method as implemented in *DADA2*. A naïve Bayesian classifier within the SILVA v138 database was applied for taxonomic assignments of obtained nonchimeric amplicon sequence variants (ASVs). The *phyloseq* package (v1.48 [75]), was further used for data processing. ASVs assigned to chloroplasts, mitochondria, and unassigned ASVs at the phylum level were removed from the dataset, and cleaned using the *MicroViz* package (v0.12.5; [76]). Prevalence-based contamination was assessed using controls with the ‘*decontam*’ R package (v1.16) and the default P threshold of 0.1 [77].

## Astrovirus prevalence

First, we calculated the average prevalence of AstV among 434 virus-screened *C. perspicillata* for each sampling site. We compared mean prevalence (square-root transformed to meet normality assumption) using a one-sided t-test with landscape (continuous forests vs. forested fragments in an agricultural matrix) as explanatory variable.

### Gut microbial composition and alpha-diversity

We calculated the gut microbial alpha diversity indices, the number of observed ASVs, Shannon index and Faith’s phylogenetic diversity, after rarefying the data to 5000 sequences per sample. Rarefying removed 35 samples with fewer than 5000 reads (final sample size: *n* = 199; [78]), although unrarefied data yielded comparable results (Supplementary Fig. 1). To analyze the effects on alpha diversity metrics (Faith’s PD and Observed ASVs were log-transformed to meet normality assumptions), we used linear mixed-effects models (LMERs) using the *lme4* package (v1.1-37; [79]) including AstV-infection (positive vs. negative), age (subadults vs. adults), sex (male vs. female), season (wet vs. dry), and landscape (continuous forests vs. forested fragments in an agricultural matrix) as explanatory variables, while still accounting for sequencing depth and setting sampling site as random effect. Since a previous study revealed that AstV infections influenced the gut microbiome dependent on host age [28], and we aimed to unveil potential interactions between AstV-infection and landscape differences, we included both two-way interactions in each model, and back-selected.

### Gut microbial beta-diversity

Microbial beta diversity distances (i.e., Bray-Curtis, Jaccard, Aitchison, weighted and unweighted Unifrac) were calculated from rarefied ASV data and ASVs agglomerated to genus level (since lower level taxonomic assignments are often more reliable for wildlife 16 S data). The gut microbial dissimilarity was compared using permutational analyses of variance (999 permutations) encoded in the *vegan* package (v2.7-2; [80]). We used the same explanatory variables as for the LMERs and stratified by sampling site. Principal coordinate analyses (PCoA) were performed to visualize the pattern of separation between the various categories of samples.

### Differential abundance analysis

Finally, we performed an analysis of compositions of microbiomes with bias correction at ASV and genus levels using both AstV infection status and landscape as explanatory factors [81]. To avoid spurious results, we removed ASVs or genera prevalent in fewer than 10% of samples, as recommended [82], and applied false-discovery rate corrections according to the Benjamini-Hochberg procedure [83].

## Results

### Astrovirus prevalence and microbiome composition

**Fig. 2:**
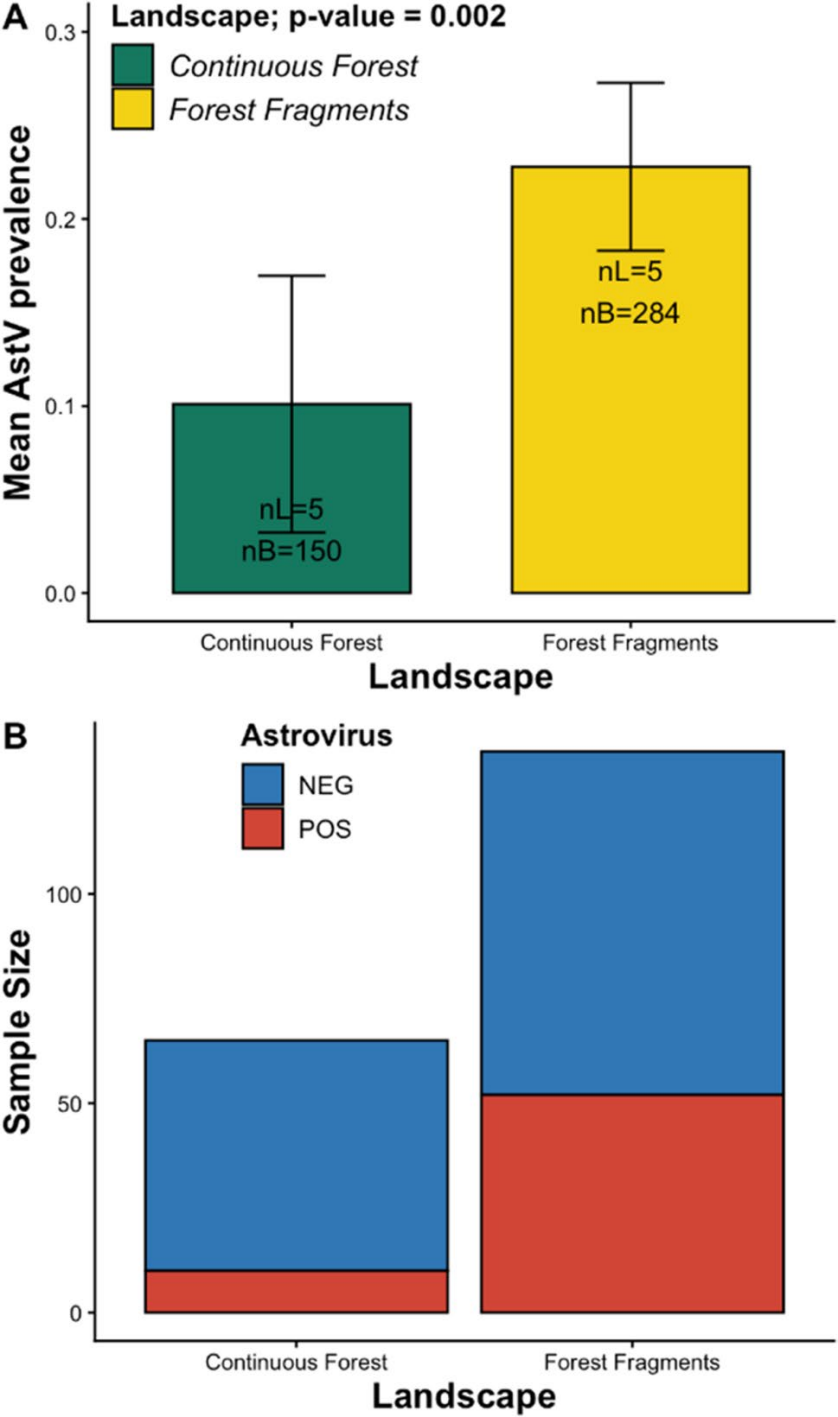
Astrovirus prevalence across landscapes. **A** Mean AstV prevalence (95% CI) captured either in continuous forest sites or forest fragments surrounded by agricultural matrix, and **B** sample sizes for the subset for which the gut microbiome was sequenced after rarefying to 5.000 reads (AstV-negative = blue; AstV-positive = red). nL and nB indicate sample size in terms of landscape and bats respectively

A total of 80 out of 434 screened *C. perspicillata* tested positive for AstV infection (Supplementary Table 1). The prevalence of AstV infections was higher in the forest fragments surrounded by agricultural matrix (mean 22.8%, 95% CI: 18.3–27.3; t-value = 4.3, *p* = 0.002; Fig. 2A) than in the continuous forest (mean 10.1%, 95% CI: 3.2–17.0). After initial filtering, 7,188,339 sequence reads of the V4 region of the bacterial 16 S ribosomal RNA gene remained from 234 fecal samples of *C. perspicillata*. On average 17,575 (range: 1,945–110,054) high-quality reads per sample were obtained after taxonomic assignments. Rarefying to a representative number of 5,000 reads resulted in the removal of 35 samples, leaving 137 AstV-negative and 62 AstV-positive *C. perspicillata* for further analysis (Fig. 2B).

**Fig. 3:**
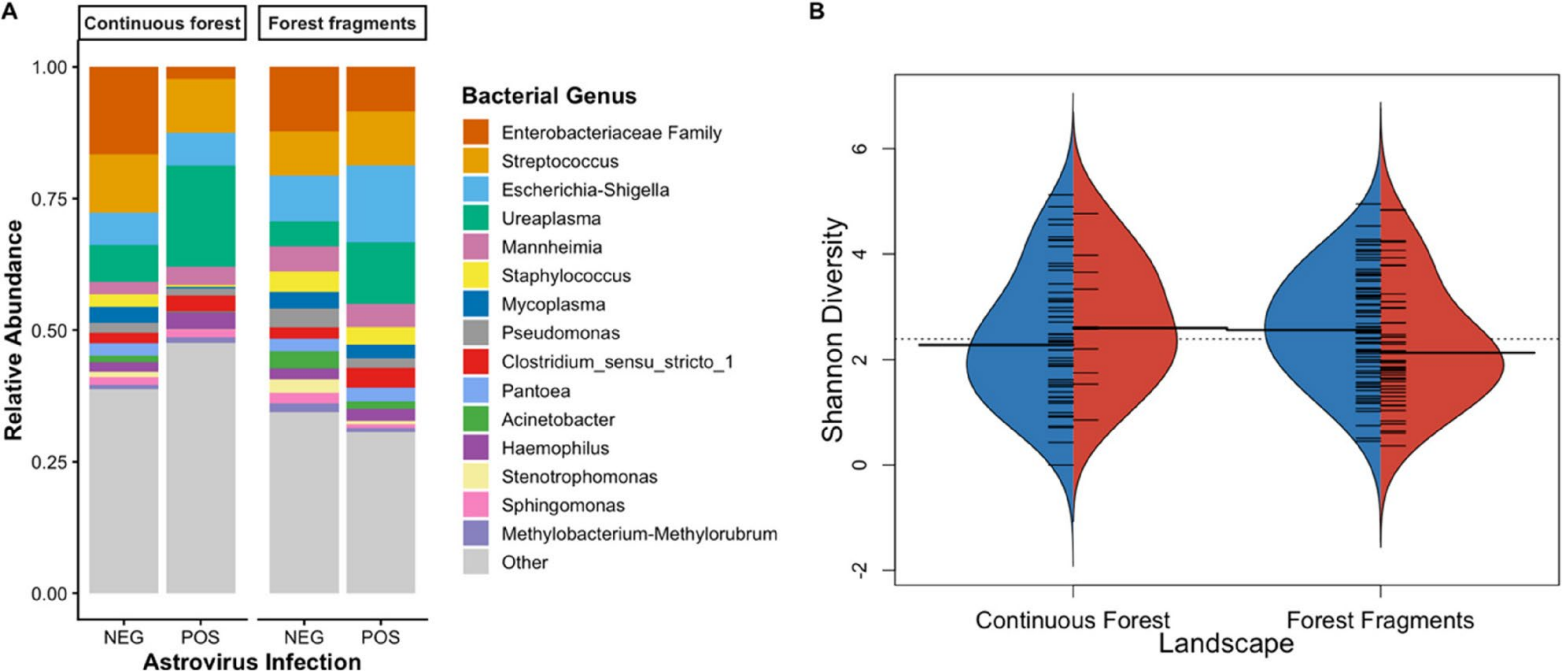
Gut microbial composition and diversity. **A** Gut microbial composition and (**B**) alpha-diversity (Shannon Diversity Index) according to Astrovirus infection status (AstV-negative = blue; AstV-positive = red) and landscape. Bacterial genera contributing less than 1% of reads are grouped as ‘Other’. In the bean plot, the dashed line denotes the overall median, the longer solid line indicates group median, and shorter solid lines depict individual samples

The dominant phylum was Pseudomonadota (mean 49.9% ± 28.9 standard deviation). At family resolution, Enterobacteriaceae (mean 21.5% ± 29.4 SD) made up the highest proportion of the microbial community, followed by Mycoplasmataceae (mean 10.7% ± 22.1 SD) and Streptococcaceae (mean 10.1% ± 15.4 SD) (Fig. 3A).

### Effects of AstV infection and landscape on alpha and beta diversity

Neither landscape differences nor infection status significantly affected any of the alpha diversity metrics in *C. perspicillata* (Supp. Table 1). There was a very weak tendency for a higher Shannon value in *C. perspicillata* positive for AstV in continuous forest, while lower values for AstV-positive individuals were recovered among the forest fragments (infection-landscape interaction: F_1,191_=2.83, *p* = 0.094; Fig. 2B). Subadult *C. perspicillata* had a lower Shannon diversity (F_1,191_=4.52, *p* = 0.034), but no interaction with age was retained in the models (Supp. Table 2). Observed ASV richness increased with sequencing depth in spite of rarefaction (Supp. Table 1).

**Fig. 4:**
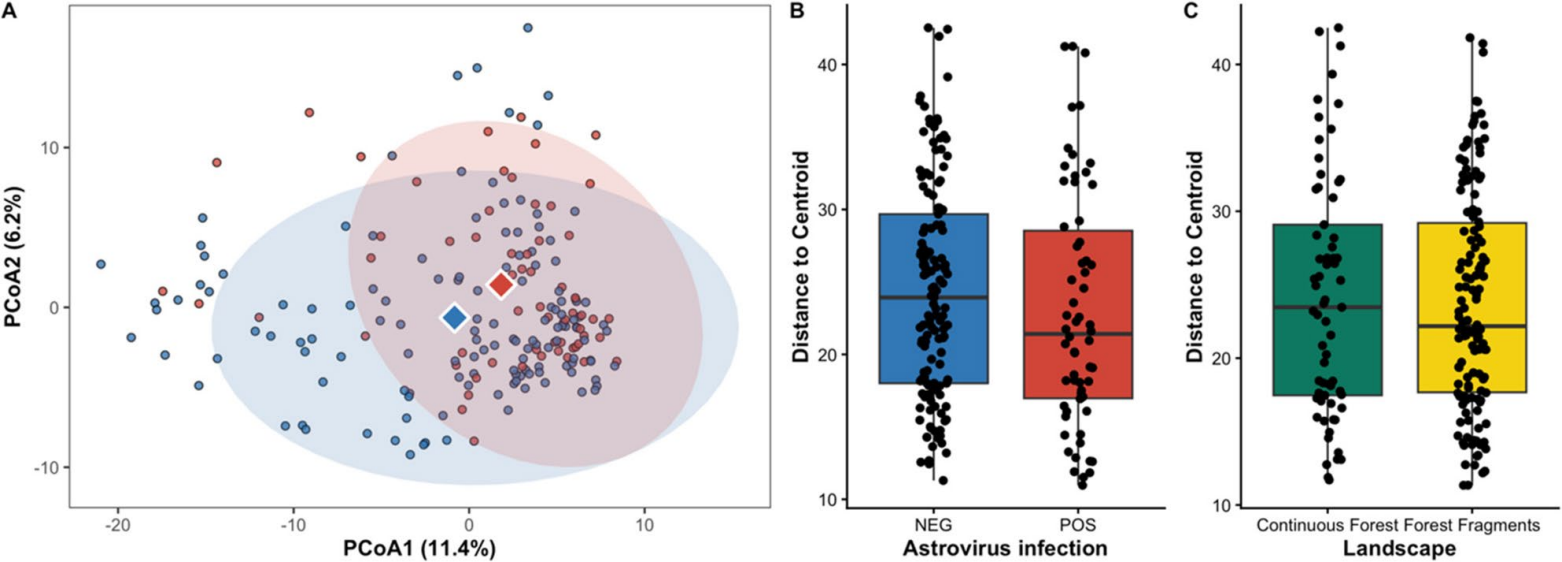
Inter-sample diversity differences between Astrovirus infection status. **A**) Principal coordinate analysis of the gut microbial beta-diversity (calculated as Aitchson distance from microbial abundances agglomerated to genus) and **B**-**C**) distances to centroid in relation to infection status or landscape. Dots are colored by infection status (AstV-negative = blue; AstV-positive = red) and statistically significant centroid differences are depicted as diamonds

AstV infection shifted the beta diversity centroid in all distances except for weighted Unifrac (Supp. Table 3). This implies that the infection substantially affects microbial community composition and, to a lesser extent, structure (Fig. 4A). A weak landscape effect was only found in Aitchison distance calculated from ASV matrix (R2 = 0.010; *p* = 0.023), but the interaction between AstV infection and landscape was never significant. Sequencing depth affected particularly distances that weighted microbial abundances based on reads (Supp. Table 3). Age did not impact beta diversity (Supp. Table 3). Beta dispersion did not differ between infection status (F_1,197_=0.14, *p* = 0.246; Fig. 3B) or landscape (F_1,197_=0.07, *p* = 0.780; Fig. 4C).

### AstV infection is associated with shifts in the relative abundance of genera and ASVs

**Fig. 5:**
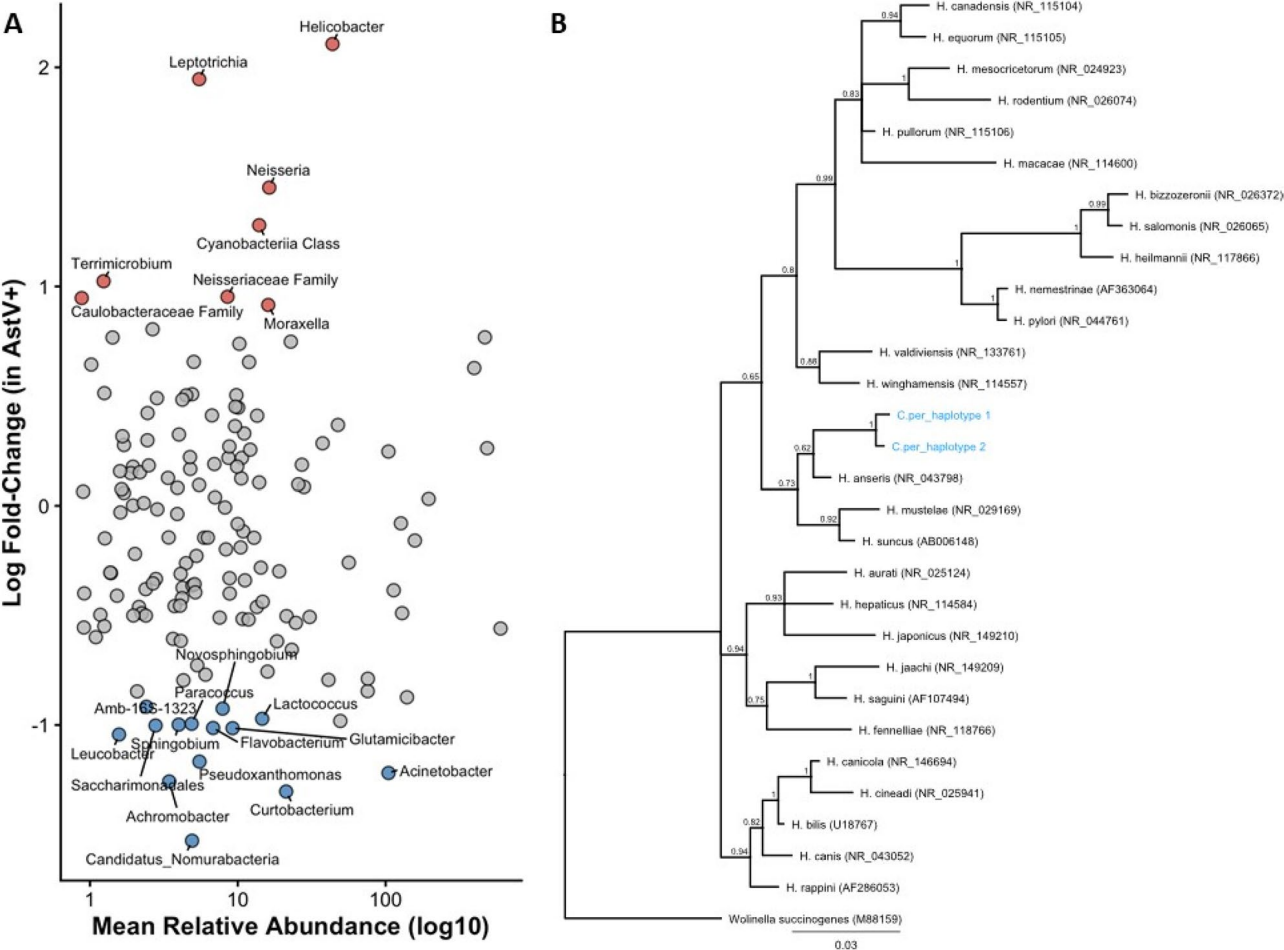
Differentially abundant bacterial genera and phylogenetic relationship of *Helicobacter*. (**A**) Differential abundance plot depicting mean relative abundances and log fold changes in bacterial genera (prevalence > 0.1) in uninfected versus AstV-positive *C. perspicillata.* (**B**) phylogenetic relationship of the two *Helicobacter* haplotypes (blue) identified in *C. perspicillata* relation to other known *Helicobacter* species (GenBank accession numbers upon publication)

In total, 29 ASVs and 23 genera were found to be differentially abundant between AstV-positive and AstV-negative C. *perspicillata* (Supp. Tables 4, 5): The genera *Helicobacter*, *Leptotrichia*, *Neisseria* and *Moraxella* containing species pathogenic to humans or animals, were enriched in AstV-positive *C. perspicillata*, for instance (Fig. 5A). Based on these results, we amplified an approximately 1200 bp long sequence in 40 randomly selected samples per landscape using a *Helicobacter*-specific primer pair [84], and aligned the resulting sequences to sequences from 27 *Helicobacter* species and *Wolinella succinogenes* as an outgroup (see Supplementary Material for more details). Two *Helicobacter* haplotypes (*C.per*_haplotype 1 and *C.per*_haplotype 2) were identified and aligned most closely to *Helicobacter anseris* (Fig. 5B).

By contrast, bacterial genera often beneficial to hosts, such as the lactic-acid-producing *Lactococcus*, the sugar-fermenting order of Saccharimonadales, and the aromatic-compound-degrading genus of *Novosphingobium*, decreased in abundance in AstV-positive bats. Although landscape effects were minimal on the whole community, certain bacteria were still more abundant in continuous forests than in forest fragments independent of the infection status (e.g., *Paenibacillus* and *Actinomyces*; Supp. Tables 4, 5).

## Discussion

Bats are known to host numerous virus families with zoonotic potential [47] and harbor a particularly high diversity of AstVs, playing a key role in AstV ecology and evolution [47–49]. In our study, the Seba’s short-tailed bats (*C. perspicillata*) appeared healthy and showed no overt clinical symptoms (e.g., weakness), yet they were found to be infectd with AstVs. Based on previous studies [28, 60, 85, 86] and public databases [87], this is the first report of AstV infection in this species. AstV prevalence was higher in bats captured in forest fragments than in old-growth lowland rainforest sites in Panama. Furthermore, gut microbial community composition – a proxy for host health [88–90] – differed between AstV-negative and AstV-positive bats, suggesting that enteropathogenic viral infection influences the taxonomic diversity of the microbiome. A decline in gut microbial homeostasis during infection may increase the risk of co-infection [38], and enhance viral (and bacterial) shedding [43, 91], posing a potential One Health concern. Consistent with this, two novel *Helicobacter* haplotypes closely related to *H. anseris* were detected in AstV-positive *C. perspicillata*.

Human encroachment into nature is likely the main driver behind the current extinction crisis [92, 93]. Additionally, research over the last decades has shown that land-use changes further increase the risk of disease spillover from wild animals into livestock and humans [8, 9, 16] and spillback into wildlife [94]. We document that AstVs were twice as likely to be detected in *C. perspicillata* captured in forest fragments embedded in agricultural landscapes than in individuals captured in continuous forests. This is unlike findings from the Bornean rainforest where AstV infection likelihood in insectivorous bats captured in actively logged, fragmented, and recovering forest sites did not differ [29]. Similarly, landscape differences affected neither AstV prevalence [28] nor host abundance of the canopy frugivore *A. jamaicensis* [58]. By contrast, the understory frugivore *C. perspicillata*, which consumes fruits of early successional plants [62, 63], such as pepper plants (Family: *Piperaceae*, genus: *Piper*) – common in edge habitat surrounding forest fragments – is four times more likely to be captured in forest fragments than continuous forest sites [58]. According to telemetry data, *C. perspicillata* has small home ranges compared to other frugivorous phyllostomids, and likely roosts close to ample food sources [66, 95, 96]. Therefore, *C. perspicillata* densities may be higher in the edge habitat around forest fragments, suggesting that AstV transmission could be density mediated [11, 97, 98]. This means that land-use change is still at the root of the rise in disease prevalence, because regionally patchy resources become more localized with fragmentation, amassing a higher number of hosts and thus increasing transmission risk.

The gut microbial community of AstV-positive *C. perspicillata* was shifted independently of whether the bat was captured in forest fragments or old-growth forest sites. This implies that land-use differences do not amplify disease-mediated changes to the gut microbial community. The gut microbial community was also different in *A. jamaicensis* infected with AstVs although, in this case, the effect was dependent on host age [28]. While cause and effect are difficult to disentangle in naturally infected populations [38, 44, 88], AstV infections are known to alter the gut mucus barrier [26], offering a mechanism by which an enteropathogenic virus could tamper with the balance of beneficial and pathogenic gut bacteria [99, 100]. Indeed, some beneficial bacteria, i.e., the lactic acid-producing genus *Lactococcus*, adhere to the outer mucus layer surrounding host epithelial cells using pili, anchoring and mucin-binding proteins [101, 102]. A disruption of the mucus barrier may thus impact resident symbionts. While we cannot elucidate the precise mechanism, *Lactococcus* and other potentially beneficial gut bacteria, such as the aromatic-compound-degrading genus of *Novosphingobium* – identified to support the breakdown of defensive plant flavonoids [103] – declined in AstV-positive bats.

Bacterial ASVs or genera that increased in AstV-positive bats range from commensals to pathogens, including the genera *Leptotrichia* and *Helicobacter*. Many *Leptotrichia* are able to ferment mono- and disaccharides to lactic acid and are found as commensal part of healthy hosts’ oral and intestinal microbiome [104]. Yet, *Leptotrichia* can be opportunistically pathogenic and were implicated in dental decay [105], or gut dysbiosis in hosts with bacterial or viral co-infections [106, 107]. The bacterial genus most strongly enriched in AstV-positive bats was *Helicobacter*, however. *Helicobacter* are gram-negative bacteria able to penetrate the mucous lining of the gut due to their helical and flagellated morphology [108]. In humans, infections with *Helicobacter pylori* cause gastric disorders and may even induce cancer [109]. Other *Helicobacter* species circulate in livestock and wildlife, and are often associated with gastric histopathological alterations [110–112]. We identified *Helicobacter* haplotypes clustering most closely to *Helicobacter anseris*, which was described in Canada geese for the first time in 2006 [113], but has since been isolated in a number of birds from South America [114, 115]. *H. anseris* grows optimally in temperatures ranging between 37 and 42 °C [113], which makes it feasible that *H. anseris* or a closely related strain could thrive in bats, which, similar to birds, maintain elevated body temperatures during flight [116, 117]. In addition, bats and birds share similar gut microbial communities, which are unlike those of other mammals [118]. One could theorise that *Helicobacter* strains evolved in birds can infect hosts with a similar gut microbial community more easily. Yet, we lack histopathological information to determine if the *Helicobacter* haplotypes were harmful to *C. perspicillata*. Taken together, AstV infection in bats seems to replace beneficial bacteria with potentially pathogenic ones, undermining the services the host may derive from its microbiota.

In comparison with the AstV infection, land-use differences seem to matter little to the gut microbial alpha and beta diversity in *C. perspicillata*. This disagrees with findings from neotropical bats captured in Mexico [119] and Belize [120], and a temperate insectivorous species [103]. Yet, our conclusions are drawn from a much larger sample size and greater within-landscape replication. Still, how might we explain these contrasting observations? Home ranges of *C. perspicillata* are small compared to other bats [66, 95, 96], and their preferred diet is found in lowland rainforest sites and surrounding forest fragments. We speculate therefore that the reason for a negligible landscape effect is explained by their relatively stable diet across landscapes. Vice versa, distinct microbiomes along a disturbance gradient in diet generalists may actually reflect shifts in diet composition [18, 121–125]. In line with this argument, the gut microbiome of the more opportunistic *A. jamaicensis* was different in pristine forest sites but similar in urban and agricultural sites in Mexico [119]. In species with diet preferences, such as *C. perspicillata*, one of two scenarios is likely to follow land-use changes: either the species cannot find their preferred diet, declines in abundance and simply does not persist in fragmented and urbanized landscapes (as seen in many insectivorous bats [20, 21, 58]) or the species can find their preferred diet, thrives, but their microbial composition remains unchanged.

Our results fit squarely within the One Health framework by demonstrating how environmental change, disease ecology, and wildlife health intersect. Landscape differences affected the species in so far as to increase the likelihood of being infected in forest fragments surrounded by an agricultural matrix. The change in gut microbial composition from AstV infection is therefore more likely in forest fragments. The loss of beneficial and enrichment of pathogenic members may shift the gut microbial community in ways relevant to host health and transmission. This highlights the importance of recognizing species- and context-specific dynamics when assessing disease risks within a One Health framework.

## Supplementary Information

Supplementary Material 1.

Supplementary Material 2.

Supplementary Material 3.

Supplementary Material 4.

Supplementary Material 5.

Supplementary Material 6.

## Data Availability

Sequencing data of C. perspicillata can be accessed via the NCBI BioProject PRJNA715730 ( https://www.ncbi.nlm.nih.gov/bioproject/?term=PRJNA715730). Helicobacter sequences from PX830598 ( https://www.ncbi.nlm.nih.gov/nuccore/PX830598) and PX830599 ( https://www.ncbi.nlm.nih.gov/nuccore/PX830599 ). Statistical analysis and phyloseq object including metadata can be found under ( https:/github.com/DominikWSchmid/Cperspicillata_AstV_16S ).
