# Supplementary material for: Astrovirus infection alters gut microbial communities in a widespread neotropical bat across human-modified landscapes
Source: BMC Microbiol. 2026 Jul 29;26:677. doi: 10.1186/s12866-026-05455-0 (PMC13421094; doi:10.1186/s12866-026-05455-0)
Supplement: Supplementary file 6 — Supplementary Material 6. [file 12866_2026_5455_MOESM6_ESM.docx]

**Supplementary Material:**

*Sanger Sequencing of Helicobacter haplotypes*

To test the faecal samples for *Helicobacter* prevalence the 16S ribosomal RNA of Helicobacter was amplified using the *Helicobacter*-specific primer pair: C97 [5’-GCTATGACGGGTATCC-3’(276–291 forward)] and C05 [5’-ACTTCACCCCAGTCGCTG-3’(1478–1495 reverse)] [84], amplifying a sequence of approximately 1200 bp. Total PCR volume amounted to 15.0 μl, consisting of 4.5 μl water, 7.5 μl AmpliTaq Gold 360 Master Mix (Applied Biosystems, CA; USA), 0.33 mM of each primer, and 10-30 ng sample. *C. perspicillata* samples were put to 40 cycles. All PCRs were run in duplicate. Following the PCR, 5.0 μl of each product was mixed with 2.0 μl loading buffer including the DNA staining dye GelRed (Biotinum, CA; USA), and were separated by gel electrophoresis. For size reference, FastRuler Low Range DNA Ladder (Thermo Fisher Scientific, MA; USA), with DNA fragments of 1500, 850, 400, 200, and 50 base pairs, was applied with the same mixing proportion as the products.

After evaluation under UV-light, Helicobacter-positive samples, were first purified with exonuclease and FastAP Thermosensitive Alkaline Phosphatase (ThermoFisher Scientific, MA; USA) to remove all single-stranded nucleotides in order to achieve a clean sequencing reaction. The purification process consisted of incubation at 37°C for 15 minutes and then at 85°C for 15 minutes. After the purifying process, the sequencing PCR for each primer was carried out using BigDye XTerminator v3.1 Cycle Sequencing Kit (Applied Biosystems, CA; USA). The total volume was 10 μl comprised of 1.0 μl BigDye, 2.0 µl BigDye sequencing buffer, 1.0 µl primer (forward: C98; reverse: C05; 10 mM) and 6.0 μl consisting out of sample and water. The sequencing PCR products were purified using BigDye XTerminator purification kit (Applied Biosystems, CA; USA) by adding 10 μl BigDye XTerminator Solution and 45 μl SAM Solution to the PCR product and shaking it for 30 minutes at 1140 rpm. Lastly, after centrifugation, 20 μl of each supernatant was loaded onto the ABI 3130 Genetic Analyzer (Applied Biosystems, CA; USA).

**Supplementary Figure:**


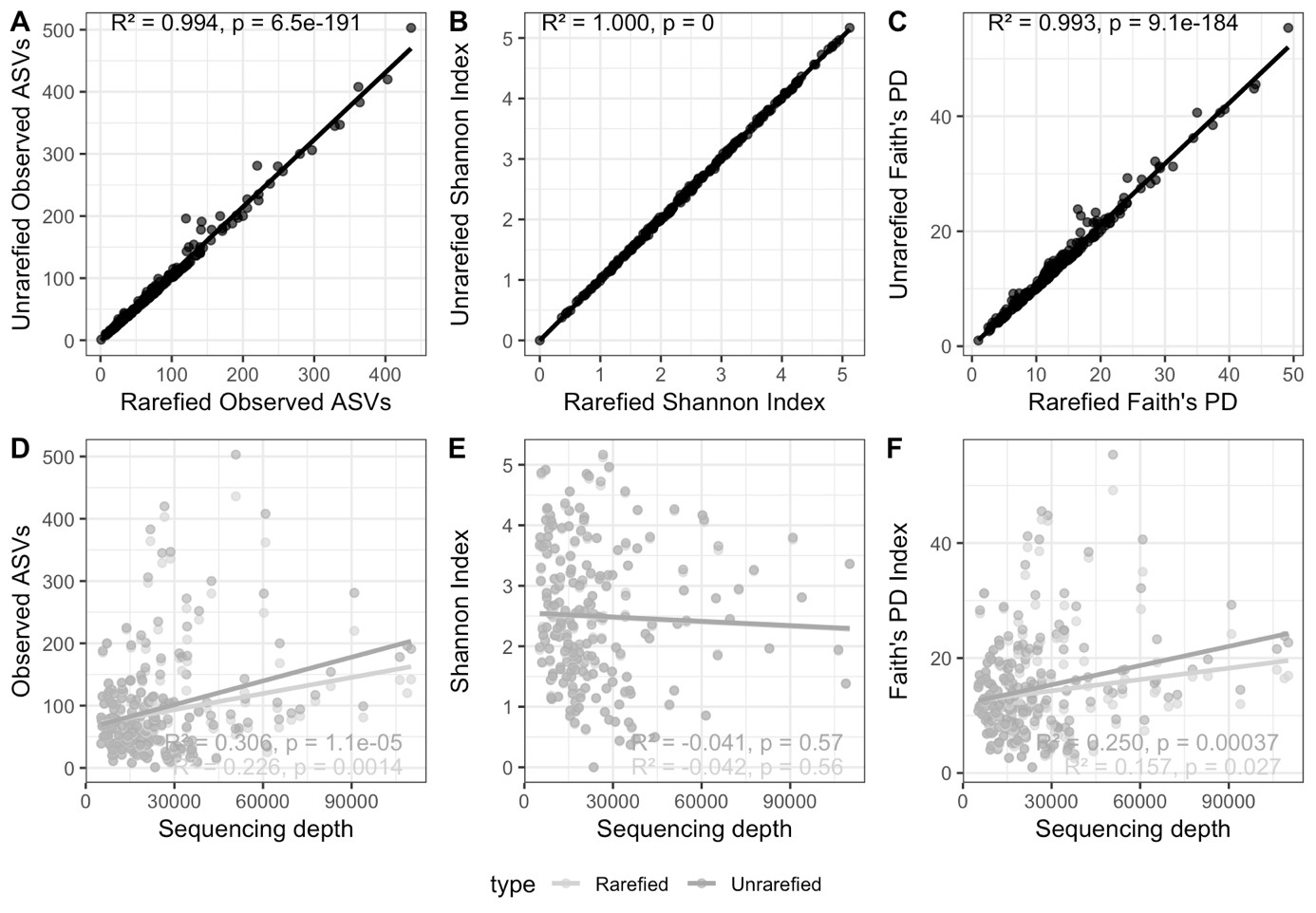


**Supplementary Figure 1.** A-C) Comparison of rarefied versus unrarefied alpha diversity metrices from samples with more than 5000 reads and D-F) in relation to sample sequencing depth.
